# Collecting and managing taxonomic data with NCBI-taxonomist

**DOI:** 10.1093/bioinformatics/btaa1027

**Published:** 2020-12-26

**Authors:** Jan P Buchmann, Edward C Holmes

**Affiliations:** Marie Bashir Institute for Infectious Diseases and Biosecurity, School of Life and Environmental Sciences and School of Medical Sciences, The University of Sydney, Sydney, Australia

## Abstract

**Summary:**

We present NCBI-taxonomist—a command-line tool written in Python that collects and manages taxonomic data from the National Center for Biotechnology Information (NCBI). NCBI-taxonomist does not depend on a pre-downloaded taxonomic database but can store data locally. NCBI-taxonomist has six commands to map, collect, extract, resolve, import and group taxonomic data that can be linked together to create powerful analytical pipelines. Because many lifescience databases use the same taxonomic information, the data managed by NCBI-taxonomist is not limited to NCBI and can be used to find data linked to taxonomic information present in other scientific databases.

**Availability and implementation:**

NCBI-taxonomist is implemented in Python 3 (≥3.8) and available at https://gitlab.com/janpb/ncbi-taxonomist and via PyPi (https://pypi.org/project/ncbi-taxonomist/), as a Docker container (https://gitlab.com/janpb/ncbi-taxonomist/container_registry/) and Singularity (v3.5.3) image (https://cloud.sylabs.io/library/jpb/ncbi-taxonomist). NCBI-taxonomist is licensed under the GPLv3.

## 1 Introduction

Retrieving and managing taxonomic information is central to many biological studies. Taxonomic information is often crucial metadata that helps in the organization of other biological datasets and represents a simple way to navigate and search the ever-increasing amount of data generated by genome-sequencing studies. Here, we present NCBI-taxonomist, a Python command line tool for the retrieval and handling of taxonomic information from the ‘Taxonomy Database’ (Sayers *et al.*, 2020) available at the National Center for Biotechnology Information (NCBI). The taxonomic information retrieved is stored as a cladogram based on phylogenetic concepts and encodes hierarchical relationships, in contrast to a standard phylogenetic tree that depicts evolutionary relationships.

Each node in the cladogram is assigned to an unique integer, the taxid, that can be used to retrieve corresponding information, including the scientific name, rank and parent node, among others. Conveniently, taxids are commonly shared between life science databases. For example, taxids from the NCBI Taxonomy Database fetch the same taxa from Uniprot (UniProt Consortium, 2019). The taxonomic information from the Taxonomy Database can be queried and retrieved via Entrez (Sayers *et al.*, 2020). This enables efficient and specific data queries because the taxid automatically links to related information in Entrez databases. For example, the Entrez taxid for *Homo sapiens* is 9606. Every dataset in an Entrez database, for example nucleotide sequences, metagenomic data or assemblies, containing human data, contains this taxid as its metadata and can be found using this taxid in search queries. Therefore, knowing the taxid can help to search efficiently for data related to an organism or taxonomic rank. Taxids in the Entrez Taxonomy Database are assigned by the NCBI taxonomy group and therefore not globally unique, but widely shared among different databases, for example Uniprot.

The Taxonomy Database can be queried via Entrez to map taxonomic names to taxids and vice versa, link taxids to different entries, obtain subtrees (i.e. all descending taxids for a specific taxid) and to construct more advanced taxonomic queries using boolean operators. These queries can be performed on the NCBI website or via the E-Utilities (Kans, 2020). The former approach is unfeasible to automate when performing multiple queries, while the latter requires some form of scripting to overcome downloading limitations and to extract the required information. NCBI-taxonomist is a command-line tool that can be integrated into scripts when required and uses entrezpy (Buchmann *et al.*, 2019) to communicate with Entrez, thereby facilitating the interaction with Entrez.

Existing software that requires taxonomic data, for instance ETE3 (Huerta-Cepas *et al.*, 2016), is specialized for phylogenetic analysis and requires the user to download the whole taxonomic database from NCBI’s ftp server in advance when handling taxonomic data from NCBI (https://ftp.ncbi.nih.gov/pub/taxonomy/). The current size of the compressed taxonomic database download is approximately only 50 MB (Megabytes). However, the download consists of several files, together producing ∼354 MB of data, and contains dumps of the taxonomy database tables. These files need to be updated with every new release of the Taxonomy Database. This necessarily increases complexity because of the need to parse and maintain local taxonomy databases. In contrast, NCBI-taxonomist can retrieve taxonomic information on-demand and does not require the user to download the entire taxonomic database; rather, it can locally store taxonomic information. NCBI-taxonomist implements all operations that can be performed in NCBI’s Taxonomy Database, in addition to creating user defined groups for selected taxa and a more versatile sub-tree command. NCBI-taxonomist is written in Python 3 (≥3.8) and the only dependency outside of the Python standard library is entrezpy. All interactions with Entrez, such as the use of NCBI API keys or control of the request frequencies are handled by entrezpy and described in further detail in its documentation. Because we developed and maintain entrezpy, which itself has no external dependencies, NCBI-taxonomist is less prone to suffer ‘dependency hell’ (https://en.wikipedia.org/wiki/Dependency_hell) and remain stable and available.

NCBI-taxonomist is licensed under the GNU General Public License v3 (GPL v3) and can be downloaded from PyPi (https://pypi.org/). The source code is available at https://gitlab.com/janpb/ncbi-taxonomist and the documentation at https://ncbi-taxonomist.readthedocs.io/en/latest/. In addition, a Docker container and Singularity image (Kurtzer *et al.*, 2017) for NCBI-taxonomist including jq (https://stedolan.github.io/jq/) are available. The Docker container can be obtained using the command docker pull registry.gitlab.com/janpb/ncbi-taxonomist: latest and the Singularity container using the command singularity pull library://jpb/ncbi-taxonomist/ncbi-taxonomist.

## 2 NCBI-taxonomist

**Fig. 1.**
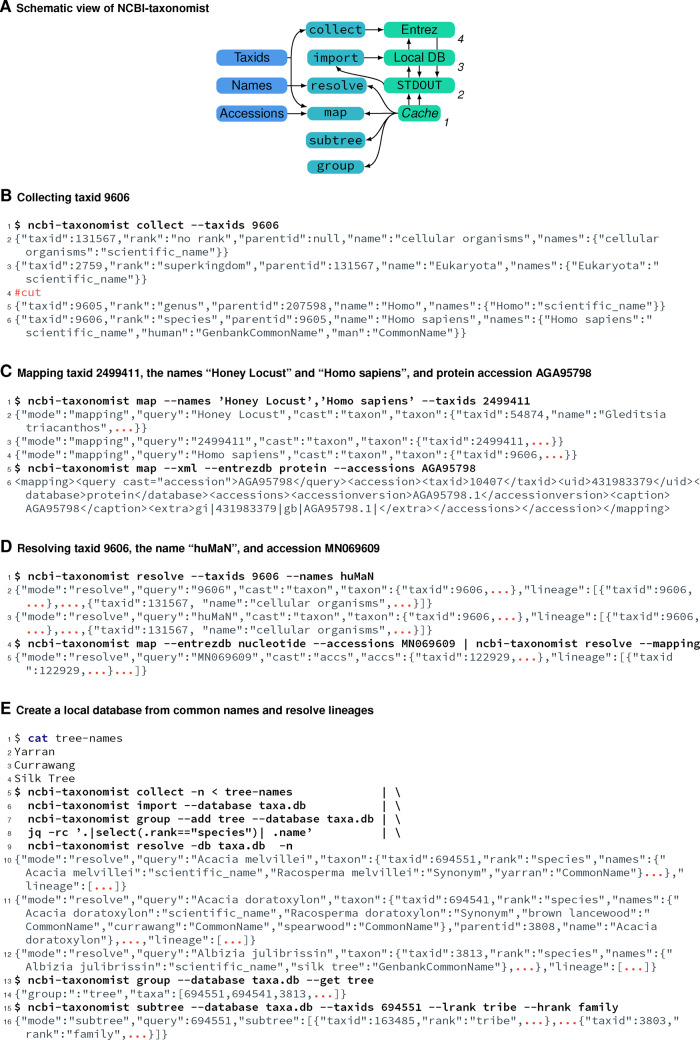
Usage examples for NCBI-taxonomist. NCBI-taxonomist commands are shown in black and the corresponding results in gray. Except for (**D**), only results in JSON are shown and partially shortened for clarity, indicated by #cut or red ellipsis. NCBI-taxonomist reports each result on one line as single JSON object or XML tree. $ indicates a terminal prompt. Examples (**B**)–(**E**) have line numbers shown on the left. (**A**) Schematic overview of NCBI-taxonomists’ operation and commands. Possible inputs are shown in the left, commands in the middle, and output or databases in the right column. The possible interactions are indicated by arrows. Cache indicates the internal cache of NCBI-taxonomist and STDOUT standard output, i.e. console output. Local DB and Entrez indicate a local and remote, Entrez database on NCBI, respectively. The numbers in the right column indicate the order NCBI-taxonomist solves queries if the corresponding arguments are given. (B) The collect command for taxid 9606. (C) Mapping accession, taxid and names. Line 5: mapping the accession AGA95798 from the Entrez Protein database in XML format. (D) The resolve command for taxid 9606, name ‘human’, and nucleotide accession MN069609. Resolving the lineages for accessions requires a mapping step. (E) Example of a more complex pipeline to create and query a local database. The cat command shows the common tree names in the file tree-names (lines 2–4). Line 5: Collecting taxa from Entrez for names in the file tree-name via standard input; Line 6: Importing taxa into the local database taxa.db; Line 7: create the group ‘tree’ in the local database for the imported taxa; Line 8: extract taxids for all species taxa using jq; Line 9: resolve the lineages for all collected species. Line 13: List all taxids in the group ‘tree’; Line 15: list all taxa between ranks ‘tribe’ and ‘family’ for taxid 694551

NCBI-taxonomist has six commands (Fig. 1A, see below), five of which can be linked via pipes on Unix-like systems to create more advanced pipelines. Results from NCBI-taxonomist queries are JSON objects and sent to standard output (Fig. 1B–E). This simplifies the writing of processing tools, for example viewers or using existing tools to process JSON data, such as jq (https://stedolan.github.io/jq/). For convenience, we also added XML output formats for each command. Because processing tab or comma separated outputs (TSV, CSV) are widely used, we show examples on the online manual how to use jq to select attributes from the JSON output and convert them into TSV or CSV. Individual NCBI-taxonomist commands can be chained together using pipes to create powerful, taxonomy-related pipelines for automated taxonomic retrieval and management; for instance, a collect command can be directly linked to create a local database and add the fetched taxa into a user specified group for later retrieval (Fig. 1A and E). The results of such pipelines can be used to create highly specific datasets for subsequent analyses. Several commands with the option to fetch data remotely from the Taxonomy Database can be used together with a local database, in which case the local query will run first. In such a situation local queries not producing a result will be tried remotely.

### 2.1 Synonyms, homonyms and spelling mistakes

Synonyms, homonyms or common spelling mistakes will be recognized by NCBI-taxonomist if they are stored in the Entrez Taxonomy database. Upper and lowercase names will be handled by NCBI taxonomist; for example, homo sapiens or HoMO SaPiEns will return data associated with the species name *Homo sapien*s, while keeping the initial spelling in the result output (Fig. 1D). If a taxonomic name is queried with known spelling mistakes known to Entrez, NCBI-taxonomist will associate the obtained result with the spelling of the given query, but only store the correctly spelled query in the local database. NCBI-taxonomist does not transform the given input names.

### 2.2 Commands

All available commands can be seen by invoking NCBI-taxonomist without any arguments, i.e. ncbi-taxonomist, and the usage for each command can be checked using the -h flag, e.g. ncbi-taxonomist map -h.

**ncbi-taxonomist collect** collects taxonomic information remotely from NCBI’s Taxonomy Database and converts each taxon into a corresponding JSON object. It accepts taxids and taxon names (known synonyms, GenBank, BLAST and common names) as input (Fig. 1B).

**ncbi-taxonomist map** maps taxids to taxon names, and vice versa. In addition, it can map sequence and protein accessions to taxids. Currently, accessions from the following Entrez databases are supported: assembly, bioproject, nucleotide and protein (Fig. 1C).

**ncbi-taxonomist resolve** resolves the lineages for taxids and taxon names (Fig. 1C). To resolve accessions, a simple pipeline comprising a mapping and resolving step can be created (Fig. 1D).

**ncbi-taxonomist subtree** extracts all lineages for specific ranks from given taxids or taxon names. If only one rank is given, only the taxonomic information for this rank is extracted. If an upper, that is, closer to the root, rank is specified, all lineages from the given rank to the lowest rank are returned. If a lower—further from the root—rank is given, all lineages from the lower rank to the root are returned. If an upper and lower rank are given, all lineages between these two ranks are returned (Fig. 1E).

**ncbi-taxonomist group** adds taxa obtained from a query to a user-specified group. Each taxon can belong to several groups. This allows the user to create specific collections based on non-taxonomic relations (Fig. 1E).

**ncbi-taxonomist import** reads query results from standard input and stores the taxa locally in an SQLite database. This allows the creation of experiment-specific taxonomy databases. It also stores the parsed taxa while printing the received input to standard output and therefore does not need to be the last step of a NCBI-taxonomist pipeline (Fig. 1E).

The versatility of NCBI-taxonomist allows it to be used for quick look-ups or to be integrated into more complex pipelines to manage or create experiment-specific taxonomic subsets. The ability to link taxonomic information to related datasets on NCBI and other life science databases via existing tools make NCBI-taxonomist a powerful approach to collecting and managing taxonomic data.

## Funding

This work was supported by the ARC Australian Laureate Fellowship [FL170100022] awarded to E.C.H.

*Conflict of Interest*: none declared.
